# Supplementary material for: Structure and activation of pro-activin A
Source: Nat Commun. 2016 Jul 4;7:12052. doi: 10.1038/ncomms12052 (PMC4932183; doi:10.1038/ncomms12052)
Supplement: Supplementary Information — Supplementary Figures 1-10 and Supplementary Table 1. [file ncomms12052-s1.pdf]

Supplementary Information

Wang et al. Structure and activation of pro-activin A

Supplementary Figure 1

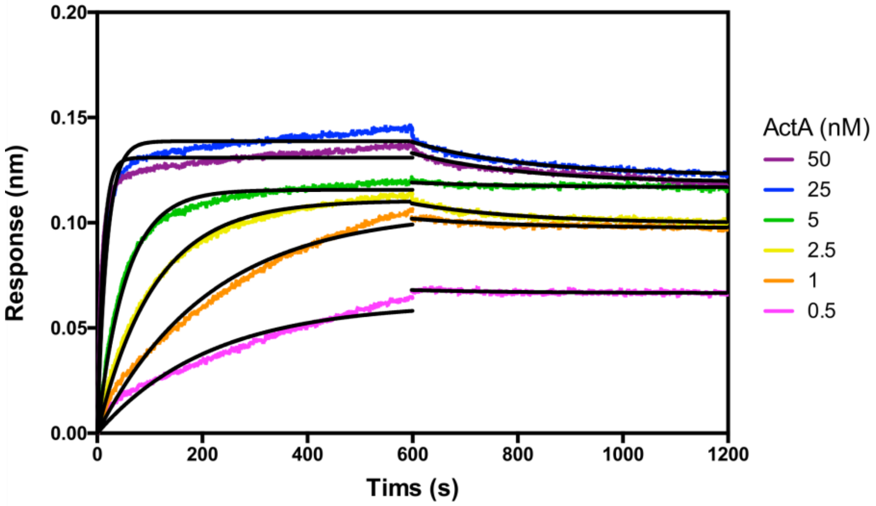

Supplementary Figure 1. Pro-mature interaction measurement using BLI. Colour traces show sensorgrams of mature activin A (in concentrations indicated on the right) binding to immobilised pro-activin A complex of which the mature domain has been stripped off using follistatin. The black lines show the data fitting to the kinetic model as described in materials and methods.

Supplementary Figure 2

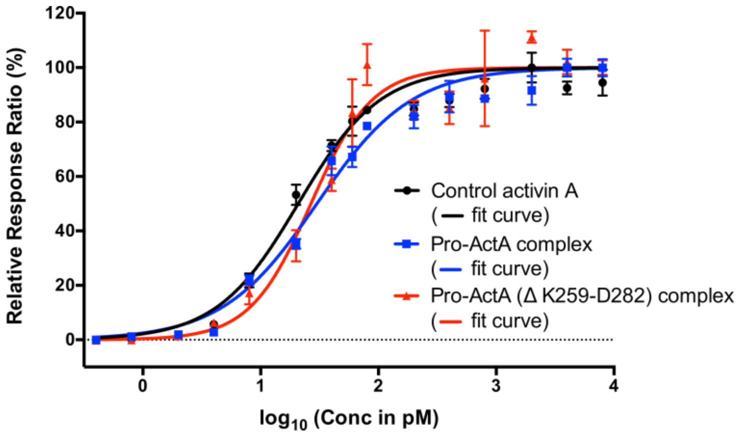

Supplementary Figure 2. Biological activity of lysine-rich loop deleted pro-activin A complex. Biological activity of cleaved pro-activin A complex as measured by luciferase-based assay in HEK293T cells. The red triangles show the data points of pro-activin A with deletion of residue between K292 and D282 and solid line shows the dose response curve. Black and blue data are for mature activin A and for pro-mature complex without deleted loop for comparison (as shown in Figure 1 of the main article), respectively. Error bars show the standard deviation from three replicate of data.

Supplementary Figure 3

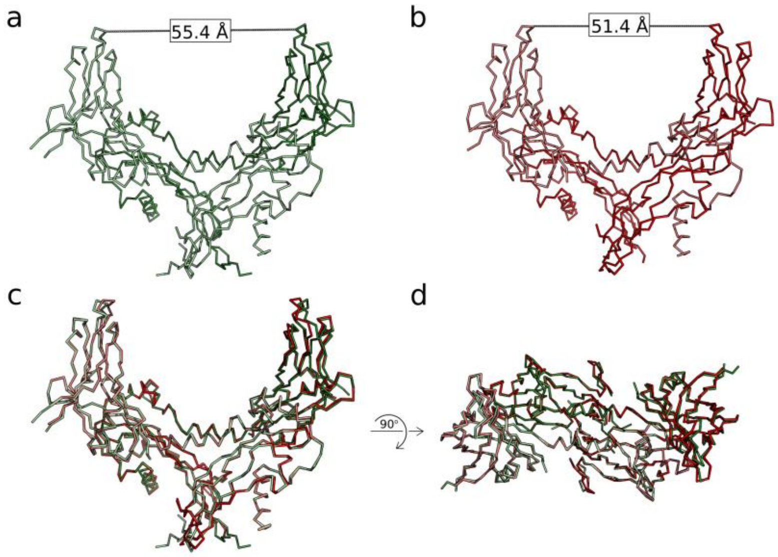

Supplementary Figure 3. Comparison of pro-activin A precursor and complex structures. (a) Activin A precursor structure (green) in dimeric form with distance between the tips of the shoulder domains shown on the dashed line. (b) Pro-activin A complex structure (red) with distance between shoulder domains as in (a). (c,d) Superpositioning of cleaved and uncleaved pro-activin A structures from the side (c) and from above (d), coloured as in (a) and (b).

Supplementary Figure 4

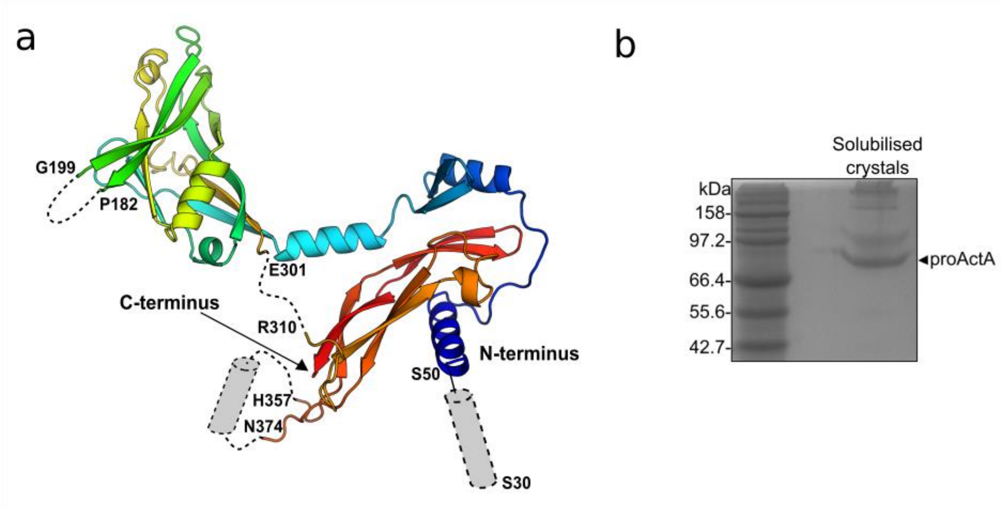

Supplementary Figure 4. Content of pro-activin A and content of pro-activin A protomer structure. (a) Structure of activin A precursor protomer, colour blue to red from N- to C-terminus, with missing parts of the crystallized protein show as dotted lines and gray helices. (b) SDS-PAGE analysis of dissolved activin A precursors crystals showing expected molecular weight for uncleaved protein.

Supplementary Figure 5

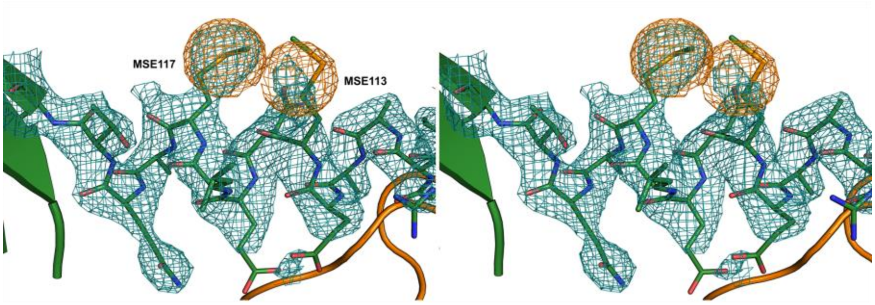

Supplementary Figure 5. Stereo image of electron density map of pro-activin A structure. The final 2Fo-Fc electron density (blue, contoured at 1σ) for the α3-helix of the pro-domain overlaid with the final, refined model. Orange density shows the anomalous difference map from selenomethionine (MSE) MAD phasing, contoured at 5σ.

Supplementary Figure 6

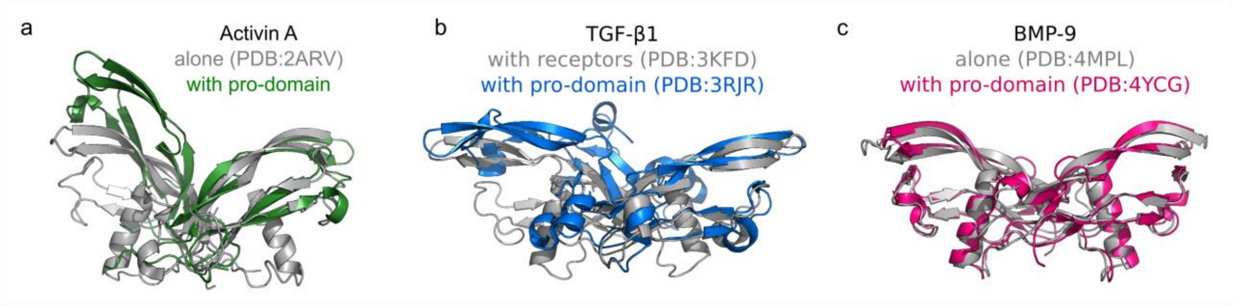

Supplementary Figure 6. Superpositioning of mature domains in pro-mature complexes and in isolation. (a) Structure of mature activin (gray) superimposed on one of the protomers of the mature domain from pro-mature complex (green, this publication). (b) Mature TGF-β1 (gray) superimposed on one protomer of the mature domain (blue) as seen in the pro-TGF-β1 complex. (c) Mature BMP-9 structure (gray) superimposed on the mature domain of the pro-BMP-9 complex (pink). PDB codes for all the structures are shown above the figures and all superpositionings are done using only the mature protomers on the right side of the structures.

Supplementary Figure 7

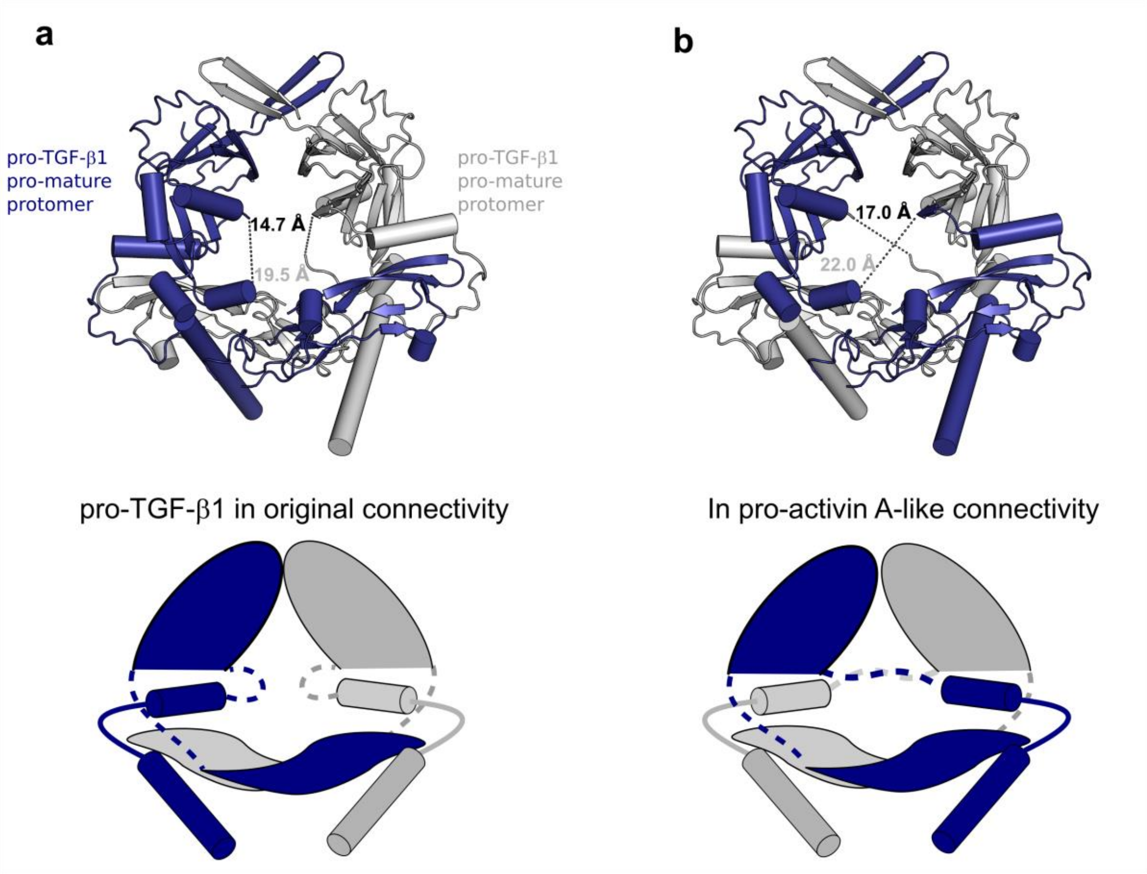

Supplementary Figure 7. Comparison of pro-mature connectivity. (a) Structure of the pro-TGF-β1 (PDB: 3RJR) coloured with one protein in darker blue and the other one in gray. Connectivity between the N-terminal forearm region and the shoulder domain of the pro-domain, as in the original publication, are indicated with dashed lines along with the distance between the terminal residues assuming that connectivity. The schematic diagram of the complex is shown underneath the structure. (b) The same structure of the pro-TGF-β1 as in (a) but coloured as if the connectivity was the same as in pro-activin A complex and again the distances between terminal residues where the density is missing shown in the figure. The schematic below shows the difference more clearly.

Supplementary Figure 8

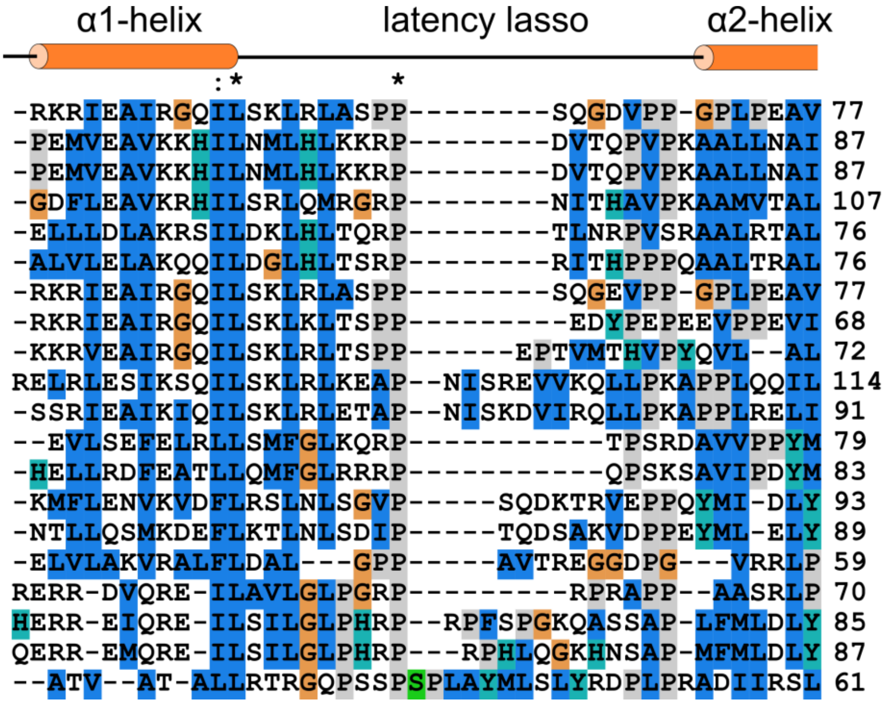

Supplementary Figure 8. Alignment of the forearm region. N-terminal forearm sequences of selected human TGF-β family proteins are aligned with pro-TGF-β (3RJR) and pro-activin A (5HLY)sequences, with the two N-terminal helices and the latency lasso marked above the alignment. The sequences are coloured based on conservation with blue indicating conserved hydrophobic residues and gray for conserved prolines. All glycines are highlighted in brown and fully conserved residues marked with asterisk above the alignment. Alignment was created and coloured in ClustalX2 program.

Supplementary Figure 9.

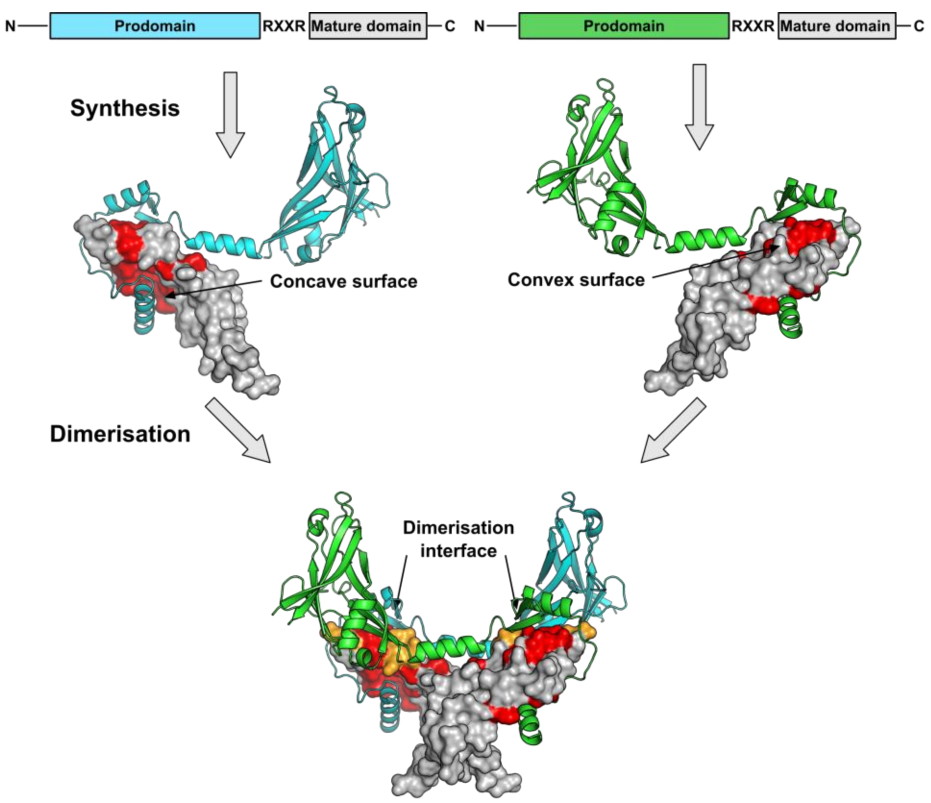

Supplementary Figure 9. Model for pro-activin A assembly. Two pro-mature activin As are synthesised (domain structure and colouring in the schemetic drawing at the top for the two proteins) and each folds into relatively compact and self-contained monomeric structures. The N-terminal helices and the latency lasso keep the mature domain (as surface model with pro-domain interfaces coloured red) in conformation that is compatible with dimerisation. The shoulder domain of the monomeric precursor, separate from the mature domain, can aid with the dimerisation process by interacting with the fingers of the other precursor and guiding the assembly of the covalent dimer.

Supplementary Figure 10.

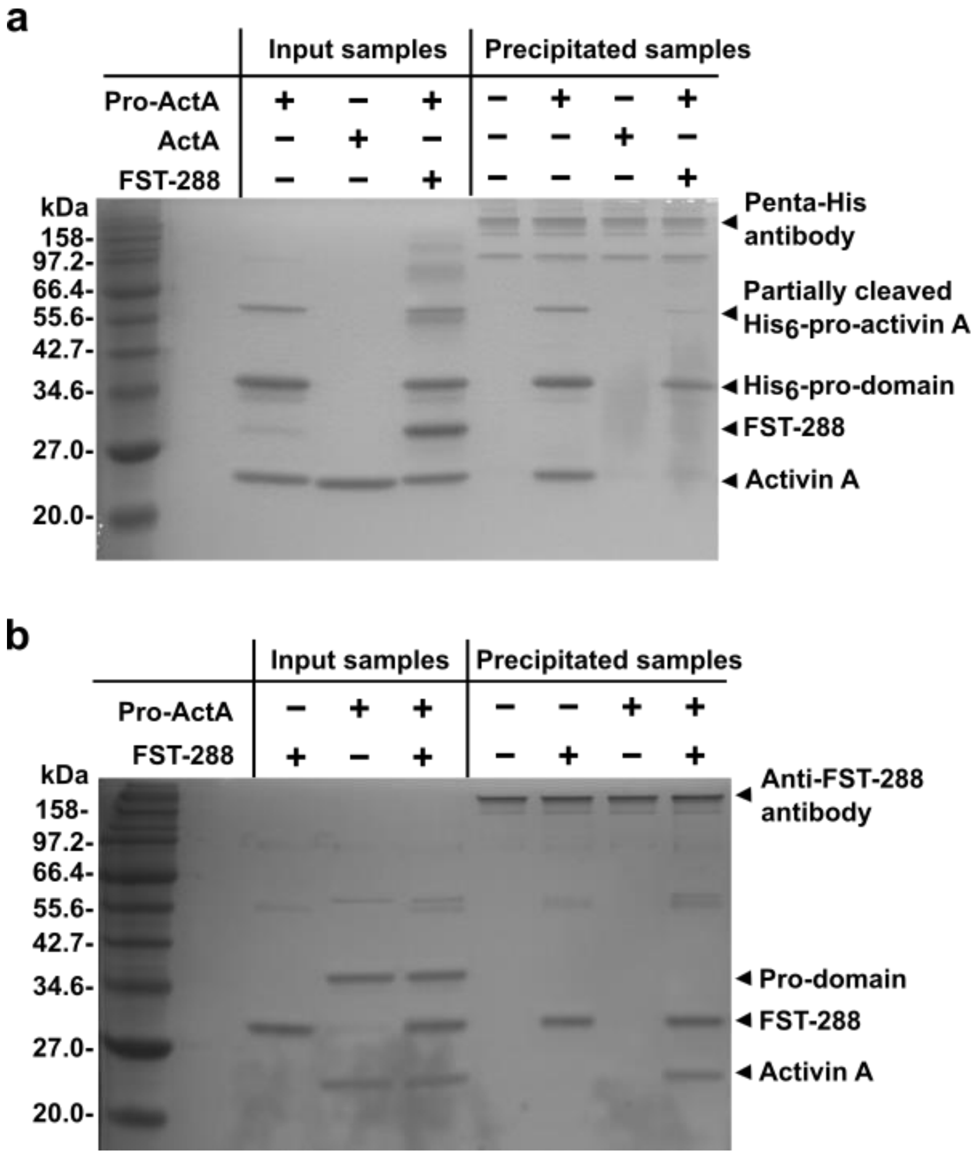

Supplementary Figure 10. Uncropped figures of non-reducing SDS-PAGE analysis. (a) Immunoprecipitation of follistatin 288 (FST-288) using penta-His antibody that binds to His6-tagged pro-activin A. (b) Immunoprecipitation of activin A using anti-FST antibody that binds to FST-288.

Supplementary Table 1. Oligonucleotides used for cloning

Cloning of expression construct

M G *S A A P D* **S** *P S* **S** *A L A*
Forward oligo 5′ TATATCCATGGGAAGTGCAGCACCAGATTCTCCATCTTCTGCACTAGCA 3′
Reverse oligo 5′ TATATCTCGAGTTATGAGCACCCACACTCCTCCACGAT 3′

Deletion of pro-domain loop residues K259-D282

*L V L L G *E E K E Q S H R P*
Forward oligo 5′ TTAGTTCTACTAGGAGAGGAAAAAGAGCAATCTCACAGACCT 3′
Reverse oligo 5′ TTGCTCTTTTTCCTCTCCTAGTAGAACTAAACTAGCTCCACTCTC 3′

Mutagenesis of furin site to HRV 3C site

E V L F Q |G P *G L E C D G K*
Forward oligo 5′ GAAGTACTATTTCAAGGACCAGGCTTGGAGTGTGATGGCAAG 3′
Reverse oligo 5′ TCCTTGAAATAGTACTTCTAGGCGATGAGGGTGGTCTTCAGA 3′

Supplementary Table 1. Oligonucletides used for cloning of the expression constructs and for the mutagenesis of the furin site and deletion of the charged loop K259-D282 used for crystallization. Underlined nucleotides show the *Nco*I and *Hind*III restrictions sites in the forward and reverse cloning oligonucleotides, respectively. Amino acid sequence are shown above the forward oligos. Asterisk indicates the position of the deleted loop and underlined sequence shows the HRV 3C protease recognition site and the cleavage site is indicated with a vertical bar. Activin A sequences are in italics in all cases. Two cysteines mutated to serines in the very N-terminus of the pro-activin A are highlighted in bold.
